# Exploring the Efficacy and Safety of Nutritional Supplements in Alzheimer’s Disease

**DOI:** 10.3390/nu17050922

**Published:** 2025-03-06

**Authors:** Paola Gualtieri, Giulia Frank, Rossella Cianci, Lucilla Ciancarella, Leonardo Romano, Moreno Ortoman, Giulia Bigioni, Francesco Nicoletti, Mario Isidoro Falco, Giada La Placa, Laura Di Renzo

**Affiliations:** 1 Section of Clinical Nutrition and Nutrigenomics, Department of Biomedicine and Prevention, University of Rome “Tor Vergata”, 00133 Rome, Italy; 2 School of Specialization in Food Sciences, University of Rome “Tor Vergata”, 00133 Rome, Italy; giulia.frank@ymail.com (G.F.); ciancarella.lucilla@gmail.com (L.C.); leonardoromanonutrizionista@gmail.com (L.R.); laplacagiada@gmail.com (G.L.P.); 3 PhD School of Applied Medical-Surgical Sciences, University of Rome “Tor Vergata”, 00133 Rome, Italy; 4 Department of Translational Medicine and Surgery, Catholic University of the Sacred Heart, 00168 Rome, Italy; rossella.cianci@unicatt.it; 5 Fondazione Policlinico Universitario A. Gemelli, Istituto di Ricovero e Cura a Carattere Scientifico (IRCCS), 00168 Rome, Italy

**Keywords:** Alzheimer’s disease, supplements efficacy and safety, microbiota, personalized nutrition

## Abstract

**Background:** Alzheimer’s disease (AD) represents one of the major challenges of modern medicine, with a growing impact on public health and healthcare systems. In recent years, dietary supplements use has been the subject of increasing interest as a complementary strategy for the prevention and treatment of the disease. **Materials and Methods**: A Review of reviews was conducted following PRISMA guidelines and REAPPRAISED checklist to evaluate the efficacy and safety of supplement use in AD. The search, performed across major scientific databases, identified 54 relevant articles, including 53 reviews and one mini-review, after applying specific inclusion criteria and removing duplicates. **Results**: The growing body of evidence suggests that some supplements may help reduce cognitive decline, inflammation, and target mechanisms behind AD. However, many of these supplements are still under investigation, with mixed results highlighting the need for high-quality research. A key challenge is the lack of data on optimal dosages, administration duration, and long-term safety, which limits clinical guidelines. Some studies have reported positive effects from specific regimens, such as curcumin (800 mg/day), omega-3 fatty acids (2 g/day), and resveratrol (600 mg/day). Other supplements, like phosphatidylserine (300 mg/day), multinutrient formulations, probiotics, vitamin E (2000 IU/day), and melatonin (3–10 mg/day), also show benefits, though study variability makes conclusions uncertain. **Conclusions**: While certain supplements show potential in mitigating cognitive decline in AD, inconsistent findings and gaps in dosage and safety data highlight the need for rigorous, large-scale trials. Future research should focus on personalized, multimodal strategies integrating targeted supplementation, dietary patterns, and microbiota-gut-brain interactions for enhanced neuroprotection.

## 1. Introduction

Alzheimer’s disease (AD) represents a new challenge, being the most common neurodegenerative disease affecting more than 30 million people aged >65 years worldwide [1]. Moreover, the prevalence of AD is expected to triple in the next two decades due to the increase in aged population [2], with a subsequent hazardous rise in costs of care [3]. In AD, there is a complex and progressive injury of the cerebral cortex due to the accumulation of β-amyloid (Aβ) plaques and neurofibrillary tangles (NFT). The causes and risk factors for the onset and the progression of AD are numerous and include the following: genetics [4]; epigenetics [5]; environmental [6]; stressors and lifestyle factors, such as diet and physical exercise; and gut microbiota (GM) [7]. These are all considered to play a pivotal role in the pathogenesis of AD.

Systemic inflammation represents the leading cause of neuroinflammation, through the activation of inflammasomes expressed by immune cells, microglia, and neurons [8]. In particular, the most implicated inflammasome in neuroinflammation is the nucleotide-binding domain (NOD), leucine-rich repeat (LRR), and pyrin domain-containing protein-3 (NLRP3) that can prompt the innate immune response through Interleukin (IL)-1β and IL-18. There is an intense trafficking among GM-related metabolites, Toll-like receptors, and inflammasomes. When an imbalance of the GM, called gut dysbiosis, occurs, it can lead to the disruption of the gut barrier (leaky gut) and the translocation of GM metabolites in the blood, which can elicit the production of pro-inflammatory cytokines.

During systemic inflammation, the pro-inflammatory mediators pass in the central nervous system by the disruption of the blood–brain barrier (BBB). The regulation of the immune response is orchestrated by the BBB and sustained by the microglia, astrocytes, and oligodendrocytes that elicit the onset of neuroinflammation through the production of neurotoxic and inflammatory factors [9].

Several GM, short chain fatty acids, and immune pathways can activate the microglia, which has been shown to be abnormal in germ-free mice [10].

Furthermore, astrocytes play a pivotal role in regulation of immune homeostasis, and they can interact with GM metabolites [11]. The activated astrocytes are not able to clear toxicants from the nervous system. In a chemical-induced colitis mouse model, researchers observed an increase in the cyclooxygenase-2 (COX-2) and glial fibrillary acidic protein within the hippocampus and the hypothalamus and a reduction in COX-2 and brain-derived neurotrophic factor in the amygdala [12].

The pro-inflammatory cytokines lead to the production of neurotoxic factors with the subsequent neuronal death [13]. GM can also yield neurotransmitters, such as Serotonin (5-HT), dopamine, and γ-aminobutyric acid (GABA).

5-HT reduction is age-related and has proven to be related to a worse cognitive performance in AD patients due to its direct action or indirect alteration of other neurotransmitters, such as catecholamines (dopamine and norepinephrine), acetylcholine, glutamate, and GABA [14]. 5-HT is implicated not only in the differentiation and death of neurons but also in the shape of Aβ and tau proteins. Drugs acting as selective 5-HT reuptake inhibitors ameliorate cognitive deficits and psychiatric behaviors in AD patients. The 5-HT receptors have been considered as drug-targets for AD [15]. Moreover, in AD, there is also a decreased levels of dopamine transmitters and receptors; dopamine agonists reduce symptoms related to frontal lobe dementia [16]. Dopamine reduction is age-related, and its receptors are correlated with cognition and memory loss. Dopamine transporters regulate the levels of dopamine and their effects play a pivotal role in cognitive impairment.

GABA is an inhibitory neurotransmitter that has been shown to decrease with age and in AD patients, and it correlates with cognitive impairment [17]. Moreover, the reduction in GABA receptors in AD has been associated with the hyperexcitability of AD patients [18].

Moreover, the GM fermentation of indigestible foods produces short-chain fatty acids (SCFA) that can pass through the BBB. Thus, it is clear that diet and the subsequent modulation of GM by foods and the related metabolites are able to modulate neuroinflammation. Moreover, it is well known that several nutrients have a beneficial effect on neuroinflammation, such as omega-3 (ϖ-3) polyunsaturated fatty acids (PUFAs) [19].

Recently, food supplements have been considered in AD due to their properties against inflammation, oxidation, and neurotoxicity [20], without reaching clear results in terms of the prevention and treatment of AD. Several food elements and supplements have been shown to improve the cognitive ability and quality of life of AD patients by regulating the immune response, production of reactive oxygen species (ROS), and neuronal protection.

Phytochemicals; polyunsaturated fatty acids; and micronutrients, such as vitamins and minerals, can prevent inflammation and subsequently Aβ production and Tau protein phosphorylation [21]. On the other hand, probiotics, by exerting a certain role on the gut–brain axis, can modulate the inflammatory response due to dysbiosis events [22].

Probiotics, such as *Lactobacilli* and *Bifidobacteria*, can enhance the levels and diversity of beneficial gut microbes through the help of prebiotics which provide substrates for their growth. Probiotics have been shown to maintain the intestinal mucosa homeostasis; avoid the alteration of gut permeability and the subsequent disruption of the gut–brain-axis; mitigate amyloid plaque accumulation, inflammatory response, and oxidative stress; and stimulate neuroprotective molecules in the brain that protect neuronal survival and differentiation [23]. Several mechanisms of action have been reported for probiotics in the modulation of the gut–brain axis and immune response, such as the production of SCFAs [24]. Firstly, SCFAs are able to ameliorate gut mucosal barrier functions and immunity. Then, they can downregulate the production of pro-inflammatory cytokines and upregulate an anti-inflammatory response. Moreover, SCFAs can modulate neuroinflammation by acting on BBB integrity and microglia activation [25]. SCFAs also present endocrine properties, such as the stimulation of glucagon-like peptide-1 (GLP-1), that can reduce neuronal death [26]. They can also stimulate the production of 5-HT and GABA.

Despite several reports, the results are contradictory and inconclusive due to the different durations of intervention, different use of diverse substances, and the patients’ features and disease severity.

For this reason, the aim of this Review of Reviews [27,28,29] is to provide the state of the art of the efficacy and safety of supplementation in the prevention and treatment of AD, with a focus on pre- and probiotics. Given the established role of supplements in modulating inflammatory pathways, gut microbiota, and immune responses, there is increasing interest in their potential to slow disease development and progression. While substantial preclinical data suggest they play a role in modulating neuroinflammation and cognitive decline, clinical evidence remains limited, and direct human applicability and the effectiveness and safety of these interventions require further investigation. This study was conducted as a Review of Reviews, as this approach allowed us to synthesize and compare results from several reviews, reducing the risk of bias associated with individual studies and providing a broader, more structured view of the available evidence. The analysis focuses exclusively on reviews to ensure a higher level of evidence synthesis, identify emerging trends, and assess the scientific consensus on the efficacy and safety of supplements in AD, overcoming the limitations of isolated clinical trials, which often have small samples and methodological inhomogeneity.

## 2. Materials and Methods

**Figure 1:**
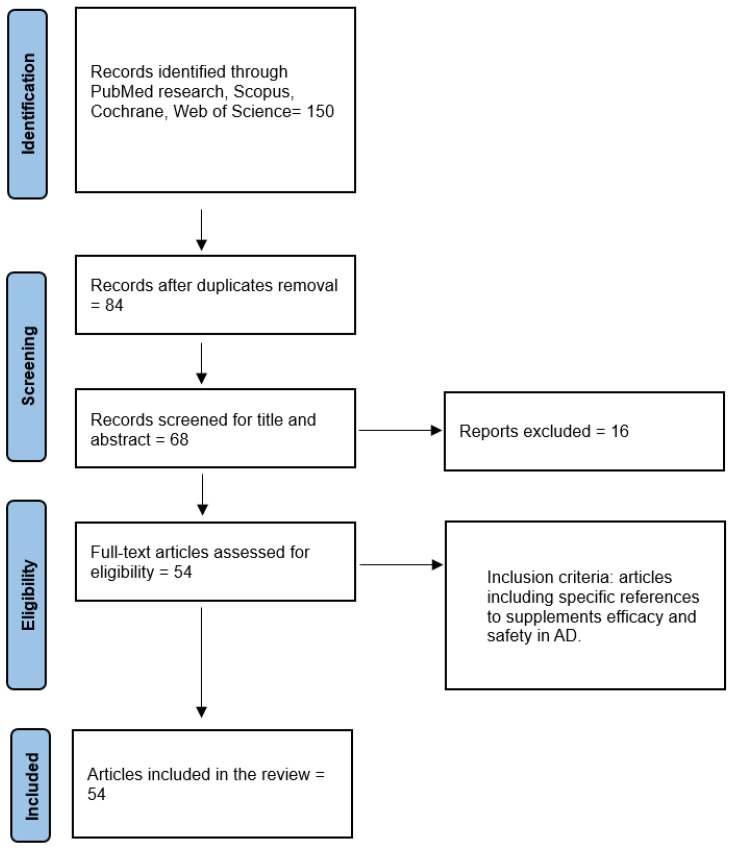
Flow-chart.

This Review of Reviews [27,28,29] was conducted from July 2024 to January 2025 using scientific databases, including PubMed, Scopus, Web of Science, and Cochrane Library. Our focus was identifying reviews related to the efficacy and safety of supplement use in AD. The filters used were “free full text”, “review”, and “5 years”. Specific keywords were employed to refine the search as follows: “efficacy of supplement” AND “AD”, “safety of supplement” AND “AD”. Only reviews published in peer-reviewed journals that explicitly mentioned the efficacy and safety of supplement use in AD in their title, abstract, or text were considered for inclusion. The search process was carried out independently by three operators following the Preferred Reporting Items for Systematic Reviews and Meta-Analyses (PRISMA) guidelines and the REAPPRAISED checklist [30]. The selection procedure began with an initial examination of the titles, followed by the abstracts, and subsequently the full texts. Duplicate articles were identified and removed after a thorough review of the titles. The selection process, reported in Figure 1, involved 150 articles. A total of 66 duplicate articles were removed, and 84 articles were initially identified as suitable. After a preliminary assessment of the titles and abstracts, 16 articles were excluded. Subsequently, after full-text screening, 14 more articles were excluded. Ultimately, 54 articles that met the criteria and were relevant to the topic were included, comprising 53 reviews and 1 mini-review.

## 3. Results

From the research conducted, 13 reviews were focused on the safety and efficacy of supplements in AD prevention, 35 reviews focused on the safety and efficacy of supplements in AD treatment, and 6 reviews focused on the safety and efficacy of prebiotics and probiotics in AD. Then, the results were divided into three different paragraphs related to the efficacy and safety of supplements in the following categories: prevention of AD, treatment of AD, and supplementation with prebiotics and probiotics.

### 3.1. Prevention

#### 3.1.1. Preclinical Studies

Catechins, particularly epigallocatechin gallate (EGCG), have demonstrated neuroprotective effects in AD models by reducing oxidative stress, modulating inflammation, and preventing protein aggregation [31,32,33]. Preclinical studies indicate that EGCG (10 mg/kg/day for one month) improved cognitive deficits and reduced ROS levels in rats with streptozotocin-induced dementia [31,32]. Additionally, epicatechin (50 mg/kg/day for four months), combined with exercise, decreased Aβ deposits and Tau aggregation in Amyloid precursor protein APP/PS1 mouse models [31]. Other studies reported improved learning and memory in aged mice at 20 mg/kg EGCG [31]. However, human dosage data remain unclear.

Ginger-derived compounds (zingerone, 6-gingerol, and 6-shogaol), in preclinical models, cross the BBB and exhibit antioxidant, anti-inflammatory, and neuroprotective properties [34]. 6-shogaol and 6-gingerol dosages are not specified, but their effects are comparable to non-steroidal anti-inflammatory drugs (NSAIDs) with fewer side effects [34].

Silymarin and *Ginkgo biloba* extracts show neuroprotective potential. However, silymarin’s bioavailability is limited, and while preclinical studies support the use of EGb 761 from Ginkgo biloba, its effectiveness in clinical settings is still under investigation [33]. No specific doses are reported for either compound.

Other polyphenols, including quercetin, kaempferol, luteolin, morin, oleocanthal, bergamot juice, and andrographolide, exhibit neuroprotective properties by modulating oxidative stress, inflammation, and Aβ pathology. Despite the promising preclinical evidence, dosages are not consistently reported, making translation to human applications challenging [32].

Moreover, several endogenous substances have been investigated for their potential neuroprotective role in AD prevention. Acetyl-L-carnitine (LC) improves mitochondrial respiration and Adenosine triphosphate production, essential for maintaining neuronal membrane potential. In vitro studies demonstrate dose-dependent neuroprotective effects against Aβ-induced toxicity, protein oxidation, lipid peroxidation, and apoptosis [33]. However, specific dosages are not reported.

L-serine has been shown to be neuroprotective in preclinical models, counteracting glutamate excitotoxicity and modulating N-methyl-D-aspartate (NMDA) receptors. It prevents neuronal apoptosis and necrosis via the phosphoinositide 3-kinases (PI3K)/protein kinase b (Akt) pathway, reduces neuroinflammation, and facilitates the recovery of cognitive and motor functions [35]. No dosage details are provided.

#### 3.1.2. Clinical Studies

Curcumin has demonstrated a dose-dependent inhibition of Aβ fibril formation and tau hyperphosphorylation, stabilizing neuronal microtubules [32,33]. It also supports macrophage-mediated Aβ clearance. Despite promising preclinical findings, only Izadi et al. [36] reported that 1 g or 4 g/die of curcumin can improve the MMSE score. However, its low bioavailability remains a limitation [33,36].

Resveratrol is neuroprotective through its antioxidant, anti-amyloidogenic, and anti-inflammatory properties, primarily via sirtuin (SIRT)-1 activation [32]. However, resveratrol has a low oral bioavailability, which limits its clinical application. No specific dosage is reported in the reviewed studies [32,33].

Coenzyme Q10 acts as an electron transporter in the mitochondrial respiratory chain and as a potent antioxidant, influencing gene expression related to cell signaling, metabolism, and transport [33]. No dosage information is provided.

Ω-3 fatty acids (eicosapentaenoic acid (EPA) and docosahexaenoic acid (DHA)) improve neurogenesis, executive functions, and learning abilities. They exert anti-inflammatory effects, reduce microglial activation and oxidative stress, and enhance mitochondrial function [33,37,38]. Deficiency is associated with memory deficits and hippocampal plasticity impairment. Supplementation studies on humans suggest a reduced risk of dementia, with benefits most evident in long-term interventions and in combination with vitamins, reporting safety doses from 230 mg to 1800 mg per day [38].

Creatine is involved in energy production and cognitive functions. While evidence on its supplementation remains controversial, some clinical studies suggest benefits, particularly under conditions of hypoxia or sleep deprivation [37]. Brain creatine levels tend to decrease with age, but differences between young and elderly individuals are not always significant. No optimal dosage for brain health is established, and further research is needed to define targeted supplementation protocols.

Citicoline seems to enhance cognitive function, especially when combined with standard treatments. However, the overall quality of clinical studies is limited, with considerable risks of bias. Although the results suggest potential benefits, more high-quality research is required to validate its effectiveness and dosages in preventing or slowing cognitive decline [39].

Taurine levels are found to be lower in human AD patients, especially in cerebrospinal fluid, and these reduced levels are associated with more severe cognitive impairment. However, research on taurine as a potential supplement and biomarker for AD remains limited [40].

While these endogenous substances show promising neuroprotective effects, significant variability exists in their reported efficacy. Importantly, only Ω-3 fatty acids have reported effective dosage ranges (230–1800 mg/day) [38], whereas for other compounds, specific supplementation protocols remain undefined. Further research is needed to optimize dosing strategies and confirm their clinical relevance in AD prevention.

Furthermore, vitamins A, C, and E exhibit antioxidant and neuroprotective effects in human AD patients by reducing oxidative stress, inflammation, and Aβ accumulation [32,38,41]. While vitamins A and E appear to support cognitive function, their efficacy in isolation remains unclear. No specific dosages are reported, but some studies suggest benefits when combined with Ω-3 fatty acids [38].

B vitamins (B6, B12, and folate) regulate homocysteine metabolism and protect against oxidative stress. Elevated homocysteine levels are linked to 12–31% of human AD patient cases [32,37]. Only one study indicated that supplementation with 0.8 mg folic acid, 20 mg vitamin B6, and 0.5 mg vitamin B12 for 24 months reduced brain atrophy by 30% in elderly individuals with mild cognitive impairment [38]. However, their efficacy appears dependent on Ω-3 plasma levels, particularly DHA [38].

Vitamin D modulates inflammation and calcium homeostasis, contributing to cognitive improvements [32,37,38]. It has been reported that a dosage of 800 IU/day for 12 months improved cognition in human AD patients with mild cognitive impairment and AD [37,38], whereas doses below 600–800 IU/day showed no significant effect [32]. Ethnic variability and the sources of vitamin D may influence outcomes.

AD patients frequently report magnesium deficiency [37]. Increased intake has been associated with a 41% reduction in AD risk, though further research is needed to confirm its therapeutic role. No specific dosage is provided.

Iron is essential for cognitive function, but its excess in AD patients promotes oxidative stress, Aβ aggregation, and neuroinflammation [37]. No specific dosage is reported.

Copper is crucial for brain development but contributes to AD progression by facilitating Aβ and tau aggregation and disrupting the BBB in clinical studies [37]. No specific dosage is reported.

Zinc is involved in Aβ metabolism, but its redistribution in AD may accelerate disease progression via the phosphorylation of tau proteins in clinical studies [32]. No specific dosage is reported.

While several micronutrients show potential neuroprotective effects, effective dosages have only been reported for B vitamins and vitamin D. The variability in study outcomes suggests that combinations with other nutrients may be necessary to achieve benefits. Further clinical trials are needed to determine optimal intake levels and confirm their therapeutic efficacy in AD prevention.

Several studies have investigated how lifestyles and dietary patterns can influence AD prevention. The Mediterranean diet (MedDiet) improves cognitive function by reducing cerebrovascular risk factors, such as dyslipidaemia, alterations in glucose metabolism, and hypertension. It also decreases inflammation, Aβ accumulation, and oxidative stress, reducing the risk of AD. MedDiet positively influences GM, improving AD-associated dysbiosis across the gut–brain axis, and it favors SCFA-producing bacteria such as *Faecalibacterium prausnitzii* and *Bacteroides cellulosilyticus*, which reduce inflammatory markers and support cognitive function. MedDiet also modulates the Lipopolysaccharide (LPS)-Toll-like receptor 4 (TLR4)-nuclear factor kappa-light-chain-enhancer of activated B cell (NFκB) pathways, reducing neuronal and intestinal inflammation and improving BBB integrity. In contrast, the metabolite trimethylamine N-oxide (TMAO), GM-derived, is associated with the increased phosphorylation of tau proteins and cognitive impairment, underlining the importance of a healthy GM in preventing neuroinflammation and neurodegeneration [32,37].

The characteristics of all articles on the safety and efficacy of supplements in AD prevention are listed in Table 1.

### 3.2. Treatment

In addition to traditional drug therapies, researchers are investigating the use of nutritional supplements and natural compounds that can offer complementary support to the treatment of slow cognitive deterioration and improve overall patient well-being. Supplementation, including antioxidant and anti-inflammatory molecules, may offer additional benefits by acting on specific physiological mechanisms.

#### 3.2.1. Preclinical Studies

Coenzyme Q10 exerts neuroprotective effects on AD by reducing oxidative stress, amyloid pathology, and brain atrophy. It mitigates mitochondrial damage, decreases Aβ production, and improves cognitive and behavioral outcomes in animal models.

Nicotinamide riboside (NR) boosts NAD+ levels, improving oxidative stress and DNA repair in AD mouse models. Cognitive improvement was assessed based on increased PARylation, a key AD hallmark, which was reduced in AD mice with NR supplementation. A daily dose of 2000 mg of NR significantly increases steady-state NAD+ levels in whole blood [42].

Several bioactive compounds found in foods and supplements have been investigated for their neuroprotective properties in AD. *Ficus carica* extracts, rich in polyphenols and flavonoids, have demonstrated antioxidant activity, Aβ reduction, and cognitive improvements in animal models, though human studies are needed to confirm efficacy and safety [43].

Similarly, licocalcones A and B from licorice and quercetin have shown potential in inhibiting Aβ aggregation and reducing oxidative stress, with taxifolin proving particularly effective in preventing cerebral amyloid angiopathy and enhancing Aβ clearance in animal models [44,45,46].

Black cumin and TQ have demonstrated significant neuronal protection in AD animal models by restoring antioxidant levels and reducing ROS, with cognitive improvement evaluated through the reduction in Aβ plaques and the inhibition of the inflammatory response via the downregulation of the NFκB pathway. However, cognitive improvements have not been assessed using specific tests, and TQ dosages of 10–20 mg/kg/day and 40 mg/kg/day for 14 days were shown to improve cognitive decline in mouse models [47].

*Sanguisorba* minor, known for its high antioxidant capacity, may help reduce inflammation caused by ROS, a key factor in AD. However, the activity of *S. minor* has been evaluated only in preclinical studies in relation to ROS production, with no efficacy tests or defined dosages conducted [48].

Hydroalcoholic extracts from *C. spinosa* fruits and leaves reduce inflammation in AD preclinical models by modulating gene expression. Seed phenolic extracts (50–200 mg/kg for 8 weeks) enhance antioxidant activity, while lowering inflammatory mediators, osteoclasts, and chondrocytes [49].

Other flavonoids, such as catechins, luteolin, myricetin, apigenin, and EGCG, have demonstrated neuroprotective effects, including the inhibition of amyloid aggregation and tau hyperphosphorylation, but human clinical trials remain limited [44,50].

Emodin, derived from *Aloe*, has exhibited antifibrillogenic, anti-inflammatory, and antioxidant properties, with significant reductions in Aβ- and tau-related damage in animal models, though further research is needed to determine the optimal human dosages [51].

Genistein, hesperidin, and cardamonin have demonstrated antioxidant effects by modulating key enzymatic pathways in preclinical models, though their safety profiles remain unclear due to the absence of dosage data [44]. Similarly, chrysin, 7,8-dihydroxyflavone, and naringenin have shown neuroprotective effects by reducing oxidative damage and promoting synaptic plasticity in experimental models [44].

Among more clinically explored compounds, bryostatin-1 (20 µg) improved cognitive function in AD patients, while scilloinositol showed benefits in animal models but not in clinical trials [52]. Cannabidiol (20 mg/kg for 8 months) demonstrated anti-inflammatory and neuroprotective effects in AD mouse models, but its mechanisms of action require further investigation [53].

Physetin has demonstrated reductions in Aβ and tau accumulation in preclinical studies [54].

Among novel compounds, sulforaphane has shown promise by activating Nrf2 and reducing neuroinflammation in animal models, though effective human dosages have yet to be determined [55].

Multifunctional chalcone derivatives have shown promising effects in AD treatment, particularly due to their ability to reduce Aβ aggregation, inhibit key enzymes, and exert neuroprotective effects. 4-hydroxy chalcone and bis-chalcone ether derivatives (20 µM) reduced Aβ aggregation from 45.9% to 94.5%, also demonstrating Cu^2+^ chelation, which may further slow plaque formation. In animal models treated with scopolamine, these compounds improved cognitive function [44]. Among dimethylamino-calcone-O-alkylamine derivatives, TM-6 (25 µM) blocked 95.1% of Aβ aggregation while also inhibiting AChE and monoamine oxidase-B (MAO-B), thereby enhancing learning and memory by improving acetylcholine and dopamine levels [44]. Antioxidant chalcone-O-carbamates, such as compound 5b, inhibited butyrylcholinesterase (BuChE) and prevented Aβ aggregation at a concentration of 3.1 µM, suggesting potential benefits in preserving cognitive function. Similarly, 16d, a scutellarein-O-alkylamine analog, effectively inhibited AChE catalytic sites, leading to memory improvements and slowed plaque formation in animal models [44]. In the ferulic acid-O-alkylamine derivative class, compound 7f inhibited BuChE and AChE, while 23c demonstrated Cu^2+^ chelation, reducing Aβ aggregation. These compounds also successfully crossed the BBB and improved cognitive function in preclinical models [44]. Other derivatives, such as coumarin-halogenated chalcones, targeted multiple enzymes (MAO, AChE, BuChE, and BACE-1). CC2 strongly inhibited MAO, while CC1 (0.69 µM) reduced MAO activity by 50%, mitigating oxidative stress and neuronal damage [44]. Hesperetin derivatives, such as compound 4f, inhibited AChE and BuChE, while also protecting neurons from oxidative stress. Another hesperetin derivative, a8, reduced Aβ 1–42 aggregation and improved cognitive function in mouse models, suggesting neuroprotective effects against neurotoxic inflammation. Additionally, 7-O-amide hesperetin derivatives (4d, 4e, 7c) prevented LPS-mediated inflammation, chelated Cu^2+^ and Zn^2+^, and exhibited antiplatelet properties against Aβ protein, further supporting their potential role in AD therapy [44]. Overall, while many of these compounds show potential in AD management, their clinical efficacy and safety remain unconfirmed due to inconsistent study results, limited human trials, and the lack of standardized dosages. Further research is needed to establish their therapeutic roles.

#### 3.2.2. Clinical Studies

Emerging evidence supports the role of nutritional supplementation in mitigating cognitive decline and modulating key pathophysiological mechanisms in AD. Among macronutrients, PUFAs have shown promising effects. In particular, α-linolenic acid (ALA) has been associated with improved neuronal development and reduced Aβ pathology. Higher ALA levels correlate with a lower risk of cognitive decline, whereas increased linoleic acid (LA) levels are linked to worse cognitive outcomes. Notably, a study on elderly individuals reported that the daily supplementation of 2.2 g of ALA for 12 weeks significantly enhanced verbal fluency, reinforcing its potential in cognitive health [56].

DHA, another essential fatty acid, has demonstrated neuroprotective effects in animal models, including anti-amyloid and antioxidant properties. However, clinical findings remain inconclusive [57]. A clinical trial involving 295 participants tested DHA supplementation but did not report significant cognitive benefits, nor did it specify the dosage used [56]. The potential synergy of ω-3 fatty acids with carotenoids such as lutein and zeaxanthin has also been explored, with some studies suggesting an improvement in memory and mood. However, the variability in study populations and dosages prevented consistent statistical significance, leaving its efficacy uncertain [58].

Phospholipids, particularly phosphatidylserine and phosphatidic acid, play a crucial role in maintaining cell membrane stability and have been investigated for their effects on AD patients. A clinical study found that supplementation with 100 mg/day of phosphatidylserine and 80 mg/day of phosphatidic acid for three months led to measurable improvements in daily activities and memory function, suggesting a cumulative and lasting benefit [58].

Beyond fatty acids, ketone bodies have gained attention due to their potential to compensate for the 10% reduction in glucose metabolism observed in AD patients. These alternative energy sources can be obtained through ketogenic diets or supplementation. Indeed, an emulsion containing 30 g/day of ketogenic medium-chain triglycerides (kMCT) was found to enhance cognitive performance, particularly in verbal fluency and memory recall tests [59].

One specific ketone body, D-β-hydroxybutyrate (βHB), appears to modulate oxidative stress and inflammation while supporting mitochondrial function. In preclinical models, supplementation with 357–714 mg/kg body weight of βHB improved mitochondrial activity and reduced neuroinflammation, suggesting its protective role against neurodegeneration. However, despite these promising findings, further clinical trials are required to establish its efficacy, determine optimal dosing, and ensure long-term safety in human subjects [60].

Vitamin E (800–2000 UI) showed promise in delaying disease progression [52], and 400 UI/die, in combination with 500–1000 mg/die of ascorbic acid, has demonstrated positive effects on cognitive performance in human AD patients, but the limited number of studies means there is not sufficient evidence to fully support their recommendation [61].

B-vitamin supplementation (B12, B6, and B9) failed to improve cognitive function in a clinical trial of 340 patients. Folic acid (400–800 µg/day for 6 months) demonstrated cognitive benefits and reduced inflammation markers (IL-6, tumor necrosis factor alpha (TNF-α), Aβ-42) [61,62,63], highlighting its potential for AD treatment [62,64].

Other compounds, such as α-lipoic acid (α-LA), appear to modulate inflammatory pathways and improve cognition [65]. Particularly, 600 mg α-LA, in combination with the acetylcholinesterase inhibitor, improves cognitive function, enhancing the MMSE, AD Assessment Scale, and cognitive subscale [66]. Similarly, vitamin D2 (800–3000 IU/day) with intranasal insulin showed mixed cognitive outcomes [58,61]. Multinutrient formulations, including Souvenaid (Fortasyn™ Connect) and a blend containing folic acid, vitamin B12, and N-acetylcysteine, demonstrated benefits in patients with mild AD, particularly in the early stages [58,67].

Additional clinical research suggests hydrogen sulfide (H_2_S) may reduce oxidative stress and Aβ accumulation. Indeed, H_2_S, through SS and the Nuclear factor erythroid 2 (Nrf2) transcription factor, activates over 200 cytoprotective genes, enhancing antioxidant defense, mitochondrial function, nicotinamide adenine dinucleotide phosphate (NADPH) regeneration, and lipid metabolism. It also promotes STAT3-induced protective proteins like Hsp90 and inhibits NFκB, reducing inflammation. However, dosages remain undefined [68].

Clinical studies suggest the potential benefits of coenzyme Q10, including short-term improvements in retinal ganglion cells, highlighting their role in combating neurodegeneration. However, no dosages were reported [69]. LC and coenzyme Q10 have been linked to neuroprotection and improved mitochondrial function, though evidence remains inconclusive [69,70]. Only Wang et al. [71] reported that 2 g/die or 3 g/die of LC helps alleviate AD symptoms, suggesting the need for further studies to confirm its effectiveness and explore its mechanisms. No cognitive improvements have been evaluated using specific tests.

*Melissa officinalis* extract, rich in rosmarinic acid, has been clinically tested for its potential to slow AD progression, and gastrodin has shown neuroprotective effects in patients undergoing cardiopulmonary bypass. However, neither has undergone efficacy testing with defined dosages [72].

Carotenoids like lycopene and phenolic compounds, including ferulic acid, ellagic acid, and caffeic acid, have shown promise in reducing oxidative stress and neuroinflammation, but studies lack standardized dosages [50]. Theaflavins and nanocarrier-based therapies are emerging as potential tools for enhancing bioavailability and BBB penetration [50].

While resveratrol (3 g/die) [73] and curcumin have shown neuroprotective properties, clinical trials have yielded inconsistent results, with resveratrol (3–5 mg/die) linked to side effects such as nausea and weight loss, and curcumin requiring further studies on its bioavailability [52,74]. Huperzine A (400 µg/day) has demonstrated cognitive benefits as an acetylcholinesterase (AChE) inhibitor in clinical trials, and homotaurine (150 mg twice daily for 78 weeks) was found to slow hippocampal atrophy [52].

Melatonin (2–5 mg for 24 weeks) showed cognitive and mood improvements in human AD patients [52,75,76].

Caffeine, which modulates beta secretase (BACE)-1 and A2A receptors, has shown potential in preventing cognitive decline, though the optimal dosage for neuroprotection remains unclear [77].

Honey and chrysin have demonstrated neuroprotective effects by modulating oxidative stress and inflammatory pathways, though dosage guidelines remain unspecified [78].

Overall, while many of these compounds show potential in AD management, their clinical efficacy and safety remain unconfirmed due to inconsistent study results and the lack of standardized dosages. Further research is needed to establish their therapeutic roles.

The characteristics of all the articles on the safety and efficacy of supplements in AD treatment are listed in Table 2.

### 3.3. Prebiotcs and Probiotcs Supplementation

The gut–brain axis has emerged as a critical player in the pathogenesis of neurodegenerative disorders such as AD.

#### 3.3.1. Preclinical Studies

Several studies have demonstrated that intestinal bacteria produce neuroactive molecules, including serotonin, GABA, and SCFAs, which exert neuroregulatory effects. Metabiotics, or probiotic-derived metabolites, have shown potential in mitigating neuroinflammation and enhancing cognitive function. Notably, dopamine produced by *Lactobacillus reuteri ATG-F4* increases IL-10 and serum dopamine levels, while metabolites from *Bifidobacterium breve A1* reduce neuroinflammation in preclinical models. SCFAs from *L. rhamnosus, L. reuteri,* and *B. fragilis* regulate anti-inflammatory pathways, suggesting they may play promising role in AD management [79].

Further evidence indicates that SCFAs such as butyrate and propionate modulate cholinergic signaling and vagal anti-inflammatory pathways, potentially alleviating AD pathology. *L. reuteri ATG-F4* has been identified as a neuromodulatory agent, increasing IL-10 and serum dopamine. Additionally, in AD mouse models, metabolites from *B. breve A1* have been shown to reduce Aβ-induced neuroinflammation via acetate production [79].

Despite these promising findings, the included studies do not provide specific dosage information, making it difficult to translate these results into clinical practice.

Microbial dysbiosis has also been implicated in AD progression, with alterations in *Verrucomicrobia* and *Proteobacteria* levels and a decline in *Ruminococcus* and *Butyricicoccus* observed in AD mice models. This imbalance is associated with the reduced SCFAs production and metabolic disruptions. Notably, the long-term use of broad-spectrum antibiotics in AD mice resulted in a decrease in Aβ deposition, suggesting that microbiota-targeted interventions may hold therapeutic potential. However, current evidence lacks standardized probiotic dosages, limiting their clinical applicability [80].

Several probiotic strains, including *Bacillus subtilis, Clostridium butyricum,* and *E. coli* (heat-labile enterotoxin), have exhibited potential neuroprotective effects through mechanisms such as amyloid degradation and the modulation of glucose homeostasis. Fermented black carrot with *Aspergillus oryzae* and *L. plantarum* has also shown promise in reducing Aβ deposition and improving cognition. However, most of these findings remain preclinical, and no explicit dosage guidelines are reported [81].

#### 3.3.2. Clinical Studies

Dietary interventions, particularly probiotic supplementation, have gained attention as accessible strategies for managing cognitive decline [82]. Strains from *Bifidobacterium* and *Lactobacillus*, commonly found in fermented foods, have demonstrated neuroprotective effects by reducing oxidative stress and neuroinflammation. Specific strains, such as *Lactobacillus plantarum MTCC1325* and *L. helveticus IDCC3801*, have been linked to improvements in learning and cognitive function, yet none of these studies provide explicit dosage recommendations. Similarly, a 12-week probiotic milk intervention containing *L. acidophilus*, *L. casei*, *B. bifidum*, and *L. fermentum* improved cognitive scores in AD patients, but without specifying the administered concentrations [81].

A few studies have provided dosing information on probiotic strains, suggesting that probiotic supplementation at 1–2 × 10^10^ CFU for 12–24 weeks may yield cognitive benefits. Specifically, *B. breve A1* supplementation at 2 × 10^10^ CFU for 16 weeks has been associated with memory improvements. Despite this, the significant variability in strains, intervention duration, and participant characteristics underscores the need for well-designed clinical trials to determine optimal dosages and long-term efficacy. Furthermore, probiotics stimulate neuroprotective molecules like GABA and SCFAs, which can cross the BBB, providing additional mechanistic support for their therapeutic potential in AD [38,83,84].

Overall, while GM represents a promising target for AD interventions, the lack of standardized dosing protocols and the reliance on preclinical models remain major limitations.

The characteristics of all articles on the safety and efficacy of prebiotics and probiotics supplementation in AD are listed in Table 3.

## 4. Discussion

The efficacy and safety of phytochemicals, endogenous compounds, and micronutrients in AD prevention and treatment are supported by promising preclinical evidence, yet their translation into clinical practice remains challenging. While these compounds exhibit antioxidant, anti-inflammatory, anti-amyloidogenic, and mitochondrial-supportive properties, a critical limitation emerges from the fact that less than half of the reviews analyzed include clinical studies on humans, while the remaining are based solely on preclinical data. This results in a significant gap between mechanistic insights and real-world applicability, raising concerns about the clinical relevance of these findings.

Catechins, curcumin, resveratrol, and flavonoids modulate oxidative stress, neuroinflammation, and amyloid metabolism, with some also influencing tau hyperphosphorylation and mitochondrial function [31,32,33,34,36,43,44,45,46,47,48,50,51,52,53,54,55,72,73,74,75,76,77,78]. However, despite their strong mechanistic support, their low bioavailability and rapid metabolism limit systemic absorption, reducing their therapeutic potential in humans [33]. Advances in delivery methods, such as lipid-based carriers and nanoformulations, may enhance their pharmacokinetics, yet clinical validation remains limited. Moreover, interindividual variability in GM composition and metabolic pathways affects their biotransformation and therapeutic response, a factor rarely considered in clinical studies.

Endogenous compounds, such as LC, coenzyme Q10, citocoline, taurine, and NR, support mitochondrial function and neuronal survival, yet the lack of standardized dosing protocols and the variability in study populations hinder their clinical applicability [33,39,40,42,69,70,71]. L-serine, known for its role in NMDA and PI3K/Akt signaling pathways, demonstrates potential in modulating excitotoxicity and reducing neuroinflammation, but optimal therapeutic concentrations and long-term safety remain unclear [35]. Among dietary lipids [33,37,38,56,57,58,61,65,66], ω-3 fatty acids (EPA and DHA) show the strongest evidence for neurogenesis, synaptic plasticity, and anti-inflammatory effects [33,37]. While effective dosages ranging from 230 to 1800 mg/day have been reported, inconsistencies in cognitive outcomes suggest that factors such as baseline dietary intake, APOE genotype, and supplement formulation may significantly influence efficacy [37]. Differences in study conditions, including participant age, disease stage, and supplementation duration, further contribute to the observed variability across trials.

The disproportionate reliance on preclinical data in the available reviews highlights a major gap in the translation of findings into clinical practice. While these studies provide essential mechanistic insights, they fail to account for the complexity of human metabolism, individual variability, and long-term safety considerations. The reviews that do include clinical trials reveal substantial heterogeneity in study designs, sample sizes, and outcome measures, making it difficult to draw firm conclusions regarding efficacy and optimal dosages.

Safety considerations are also crucial, as most of these compounds appear to be well tolerated, but their long-term effects, drug interactions, and metabolic responses remain underexplored. While creatine supplementation has shown benefits in cognitive resilience under stress conditions such as hypoxia and sleep deprivation, its role in aging-related cognitive decline remains inconclusive [37]. Similarly, vitamins B and D demonstrate cognitive benefits at specific dosages, whereas the role of antioxidant vitamins and minerals remains controversial [32,37,38,58,61,62,63,64,67], with some evidence suggesting that excessive supplementation may even be dangerous.

The translational gap between preclinical findings and clinical outcomes underscores the need for more rigorous study designs that account for bioavailability, participant heterogeneity, and methodological inconsistencies. Many trials rely on diverse cognitive assessments, complicating direct comparisons and contributing to inconsistent findings. Additionally, genetic factors, metabolic status, and GM profiles significantly influence nutrient metabolism and cognitive response, yet these aspects are rarely integrated into clinical research.

Our study presents some limitations. First, our reliance on secondary data from reviews introduces a layer of interpretative bias, as we depend on the methodologies, inclusion criteria, and quality assessments of the original reviews. Another limitation concerns the challenge of assessing bioavailability, dosage optimization, and long-term safety, as most of the reviews included focus on efficacy rather than the pharmacokinetic considerations. The translation of findings from preclinical models and small-scale human trials into real-world clinical applications remains a key challenge, and our study is limited by the extent to which reviews addressed this gap. Additionally, the included reviews may not fully account for interindividual variability, such as genetic predisposition (e.g., APOE genotype), baseline nutritional status, or GM composition, which could significantly influence responses to the supplementation. Lastly, publication bias within the included reviews poses a limitation, as studies with positive findings are more likely to be published, potentially overestimating the effectiveness of certain supplements while underreporting null or negative results.

Despite these limitations, our study provides a comprehensive synthesis of recent high-level evidence on supplementation for AD, highlighting key findings and research gaps that warrant further investigation through well-designed, long-term clinical trials.

## 5. Conclusions

Growing preclinical evidence supports the potential efficacy of certain supplements in mitigating cognitive decline, reducing neuroinflammation, and targeting key pathophysiological mechanisms underlying AD. However, due to the limited number of clinical studies, their actual impact on human AD patients remains uncertain, preventing any firm recommendations for clinical use. Indeed, a major limitation of the current body of evidence is that less than half of the reviews analyzed include clinical studies conducted on humans, while the remainder are based solely on preclinical data. This disproportionate reliance on animal and in vitro models highlights a significant gap in the translation of findings into clinical practice, as many compounds remain in the investigational stage, with inconsistent and inconclusive results. The lack of robust clinical data further underscores the urgent need for rigorous, high-quality research to validate the efficacy and safety of these interventions in human populations.

Another clinical limitation is the lack of comprehensive data on optimal dosages, the duration of administration, and long-term safety, leaving critical gaps in clinical guidelines. While some clinical studies have reported benefits with specific regimens, such as 800 mg/day of curcumin improving cognitive function, 2 g/day of ω-3 fatty acids reducing neuroinflammation, and 600 mg/day of resveratrol showing neuroprotective effects, the variability in study designs and patient responses limits definitive conclusions. Similarly, phosphatidylserine at 300 mg/day, a multinutrient formulation containing choline, uridine, and DHA, and probiotics at 1–2 × 10^10^ CFU for 12–24 weeks have demonstrated cognitive benefits in AD patients. Furthermore, studies indicate that vitamin E at 2000 IU/day may slow functional decline, while melatonin at doses of 3–10 mg/day has shown improvements in sleep and cognitive performance. However, without a stronger foundation on large-scale human trials, these findings remain difficult to generalize into clinical practice.

Personalized supplementation strategies represent a promising frontier in AD management, aligning with the principles of predictive, preventive, personalized, and participatory (4P) medicine. Considering genetic predisposition, baseline nutrient deficiencies, and metabolic status could optimize therapeutic efficacy while minimizing risks. In this context, the microbiota-gut–brain axis is emerging as a crucial yet underexplored area, with growing evidence suggesting that modulating GM may influence neuroinflammatory and neurodegenerative processes. However, the lack of clinical studies focusing on this further limits its practical application, emphasizing the need for research that bridges mechanistic insights with real-world interventions.

To maximize neuroprotection, future research should move beyond single-compound interventions and focus on multimodal strategies that integrate dietary patterns, such as MedDiet, with targeted supplementation. The promising potential of phytochemicals, endogenous compounds, and micronutrients can only be realized if the significant translational barriers are addressed. Given the current imbalance between preclinical and clinical evidence, large-scale, long-term randomized trials assessing both efficacy and safety, while accounting for interindividual variability, are crucial for validating their role in AD prevention and treatment. Bridging the gap between experimental findings and clinical applicability remains a priority to ensure that supplementation strategies can be effectively implemented in patient care.

**Figure 2:**
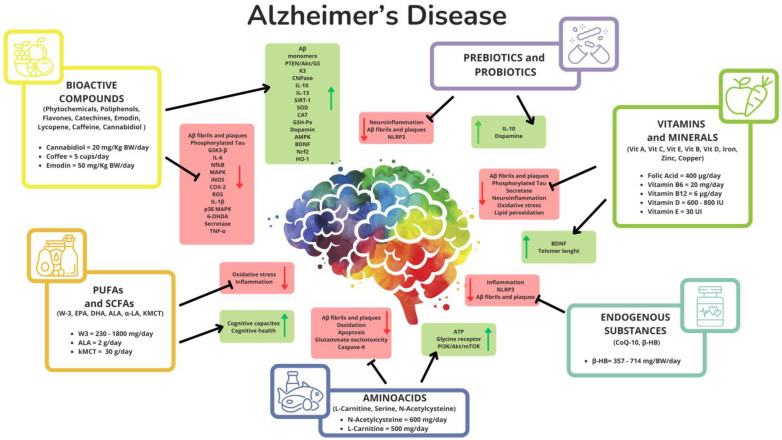
Main characteristics of all articles on the safety and efficacy of supplements in AD. Legend: black arrow, activation; black Y line, inhibition; green arrow, upregulation; red arrow, downregulation. Abbreviations: 6-OHDA, oxidopamine; w3, omega-3; α-LA, α-lipoic acid; Aβ, β amyloid; Akt, protein kinase B; ALA, alpha-linolenic acid; AMPK, AMP-activated protein kinase; ATP, adenosine triphosphate; β-HB, β-hydroxybutyrate; BDNF, brain-derived neurotrophic factor; BW, body weight; CAT, catalase; CNPase, 2′,3′-cyclic nucleotide 3′-phosphodiesterase; CoQ-10, coenzyme Q10; COX-2, cyclooxygenase-2; DHA, docosahexaenoic acid; EPA, eicosapentaenoic acid; GSH-Px, glutathione peroxidase; GSK3, glycogen synthase kinase 3; HO-1, heme oxygenase; IL, interleukin; iNOS, nitric oxide synthases; IU, international units; kMCT, ketogenic medium-chain triglyceride; MAPK, mitogen-activated protein kinase; mTOR, mechanistic target of rapamycin; NFκB, nuclear factor kappa-light-chain-enhancer of activated B cells; NLRP3, pyrin domain-containing protein 3; Nrf2, nuclear factor erythroid 2; PI3K, phosphoinositide 3-kinases; PTEN, phosphatase and tensin homolog; ROS, reactive oxygen species; SIRT, sirtuin; SOD, superoxide dismutase; TNF-α, tumor necrosis factor alpha; Vit, vitamin.

The main characteristics of all articles on the safety and efficacy of supplements in AD are represented in Figure 2.

## Figures and Tables

**Table 1:** Main characteristics of all the articles on the safety and efficacy of supplements in AD prevention.

Authors	Title	Type of Article	Date	Efficacy	Safety	Type of Study
Noll, C., et al. [31]	Catechins as a Potential Dietary Supplementation in Prevention of Comorbidities Linked with Down Syndrome	Review	2022	Catechins can protect against neurodegenerative diseases by reducing ROS production, lowering inflammation, and preventing cognitive and memory decline. The different beneficial effects of catechins have been evaluated both through the measurement of alpha and beta levels and through the Morris water maze tests.	In rats with induced dementia, an oral dose of EGCG was administered at a dosage of 10 mg/kg/day for one month. In the mouse model APP/PS1, epicatechin was administered at a dosage of 50 mg/kg/day for four months combined with exercise. In mice with accelerated aging, EGCG was administered orally at a dosage of 20 mg/kg. 10 mg/kg/day for one month of ECGC in mice models with induced dementia; to improve learning: 20 mg/kg of EGCG in mice model; to reduce plaque levels and aggregation: 50 mg/kg/die of EC for 4 months; to reduce plaque formation in mice models: 2 mg/kg/day or 6 mg/kg/day for 4 weeks; to reduce tau protein phosphorylation: combined administration of 20 mg/kg EC injections for 60 days and oral intake of 50 mg/kg for 6 months.	Preclinical studies conducted in vivo on mice and rats.
Guarnieri, L., et al. [32]	Impact of micronutrients and nutraceuticals on cognitive function and performance in Alzheimer’s disease	Review	2024	Mediterranean diet and intake of vitamins and nutraceuticals can slow cognitive decline, offering guidance for future AD treatment research. Improvements are observed in inflammatory markers and in risk factors associated with the development of AD.	Dosage not investigated.	Preclinical studies conducted in vivo on mice; clinical studies conducted in vivo on humans.
Pogačnik, L., et al. [33]	An Overview of Crucial Dietary Substances and Their Modes of Action for Prevention of neurodegenerative diseases	Review	2020	Many dietary compounds, especially polyphenols, are used as supplements for diseases like diabetes and neurodegenerative disorders. Recent reviews suggest polyphenols may improve cognitive function and reduce cardiovascular risk. Cognitive improvements have not been assessed with specific tests.	Dosage not investigated.	Preclinical studies conducted in vitro; preclinical studies conducted in vivo on mice; clinical studies conducted in vivo on humans.
Angelopoulou, E., et al. [34]	Elucidating the Beneficial Effects of Ginger (Zingiber officinale Roscoe) in Parkinson’s Disease	Review	2022	Zingerone, 6-gingerol, and 6-shogaol, the main active components of ginger, cross the blood–brain barrier and exhibit antioxidant and anti-inflammatory effects, supporting cognitive function and reducing neuroinflammation. These compounds, comparable to NSAIDs but with fewer side effects, hold promise for managing neuroinflammatory and cognitive disorders. Markers of neuroinflammation, oxidative stress, and the mechanisms regulating several transcription factors and signaling pathways, including NFκB, have been evaluated	Dosage not investigated.	Preclinical studies conducted in vitro; Preclinical studies conducted in vivo on mice.
Ye, L., et al. [35]	L-Serine, an Endogenous Amino Acid, Is a Potential Neuroprotective Agent for Neurological Disease and Injury	Review	2021	Long-term L-serine treatment may benefit AD by enhancing neurotrophic factors, promoting neural stem cell growth, and aiding brain repair. It regulates microglial polarization, reducing inflammation and neurotoxicity. Efficacy has been evaluated based on the dosage of inflammatory markers and neuronal proliferation markers.	10% L-serine for 2 months in mice models.	Preclinical studies conducted in vivo on mice.
Izadi, M., et al. [36]	Longevity and anti-aging effects of curcumin supplementation	Review	2024	Aging is a major risk factor for AD, which involves fibril formation, amyloid plaque buildup, and the loss of cholinergic neurons. Due to the side effects of current treatments, alternative therapies are needed. While curcumin may help slow disease progression and affect cognitive function, clinical trial failures could be attributed to its poor bioavailability, advanced disease stages, and differences between animal models and human patients. Cognitive improvement was evaluated using the MMSE.	1 g or 4 g/die of curcumin to improve the MMSE.	Clinical studies conducted in vivo on humans.
Kaufman, M.W., et al. [37]	Nutritional Supplements for Healthy Aging: A Critical Analysis Review	Review	2024	Supplements like creatine, magnesium, and anti-inflammatory diets as potentially beneficial for the cognitive function—all crucial for maintaining independence, reducing disability, and enhancing quality of life. Cognitive improvements have not been assessed with specific tests.	Dosage not investigated. 800 UI/die of vitamin D for 12 months improve the cognitive function in mild AD.	Clinical studies conducted in vivo on humans.
Fu, Q., et al. [38]	Supplementation and Mitigating Cognitive Decline in Older Adults With or Without Mild Cognitive Impairment or Dementia: A Systematic Review	Review	2024	Substantial evidence supporting the role of micronutrient supplementation in moderating the progression of AD. Supplements like vitamin D, probiotics, and ω-3 appear effective in slowing cognitive decline and reducing dementia risk, while vitamins A, B, C, and E show less promise. The biomarkers of Aβ plaque formation have been evaluated.	ϖ-3 supplementation with effective doses ranged from 230 mg to 1800 mg per day, in combination with vitamins. Daily supplementation of 0.8 mg folic acid, 20 mg vitamin B6, and 0.5 mg vitamin B12 for 24 months. Doses of less than 600–800 IU vitamin D produced no significant effects. 0.8 mg/die of folic acid, in combination with 20 mg/die of vitamin B6 and 0.5 mg vitamin B12 for 24 months reduced the rate of brain atrophy by 30% in the elderly. 800 UI/die of Vitamin D3 reduce the Aβ-related biomarkers.	Clinical studies conducted in vivo on humans.
Bonvicini, M., et al. [39]	Is Citicoline Effective in Preventing and Slowind Down Dementia?—A Systematica Review and a Meta-Analysis	Review	2023	This systematic review and meta-analysis on citicoline suggests that it has a positive impact on cognitive function in patients with AD, MCI, and post-stroke dementia. Citicoline appears to improve cognitive status, particularly when used alongside standard treatments. However, the overall quality of studies is low, with significant bias risks. While findings indicate potential benefits, further high-quality research is needed to confirm its efficacy in preventing or slowing cognitive decline. Cognitive functions measured by cognitive scales (MMSE, MocA, and SCOPA-cog), GCI, human vigilance, and visual working memory.	Dosage not investigated.	Clinical studies conducted in vivo on humans.
Rafiee, Z.; et al. [40]	Taurine Supplementation as a Neuroprotective Strategy upon Brain Dysfunction in Metabolic Syndrome and Diabetes	Review	2022	Epidemiological studies show that obesity and insulin resistance increase the risk of AD. Taurine levels have been found reduced in AD patients, particularly in CSF, and lower levels correlate with worsened cognitive function. Research on taurine as a potential biomarker for AD is still limited. Cognitive improvements have not been assessed with specific tests.	Dosage not investigated.	Clinical studies conducted in vivo on humans.
Ozawa, H., et al. [41]	Effects of dietary food components on cognitive functions in older adults	Review	2021	Vitamins C, E, and A play a crucial role in protecting against oxidative stress, which is linked to aging and AD. Vitamin E, especially α-tocopherol, is vital for brain health, and its antioxidant properties are linked to aging and dementia. Vitamin A also supports brain function, though it has received less focus in AD research. High levels of vitamins A and E may contribute to longevity. Cognitive improvements have not been assessed with specific tests.	Dosage not investigated.	Clinical studies conducted in vivo on humans.

Abbreviations: AD, Alzheimer’s disease; Aβ, amyloid beta; APP/PS1, Amyloid-beta precursor protein/Presenilin 1; CSF, cerebrospinal fluid; EC, epigallocatechin; EGCG, epigallocatechin gallate; GCI, global cognitive impairment; IU, International Units; MCI, mild cognitive impairment; MMSE, Mini Mental State Examination; MocA, Montreal Cognitive Assessment; NFκB, nuclear factor kappa-light-chain-enhancer of activated B cells; ROS, reactive oxygen species; SCOPA-cog, SCales for Outcomes in PArkinson’s disease-COGnition; ω-3, omega-3.

**Table 2:** Main characteristics of all the articles on the safety and efficacy of supplements in AD treatment.

Authors	Title	Type of Article	Date	Efficacy	Safety	Type of Study
Mehmel, M., et al. [42]	Nicotinamide riboside—the current state of research and therapeutic uses	Review	2020	NR increases NAD+ levels, improving oxidative stress and DNA repair in AD mouse models. NR can also reduce neuroinflammation, amyloidogenesis, and other aspects of AD neuropathology, enhancing hippocampal synaptic plasticity and cognitive functions. Cognitive improvement was evaluated in relation to increased PARylation, another hallmark of AD, which could be decreased in AD mice with NR supplementation.	2000 mg/die of NR can significantly increase steady-state, whole-blood levels of NAD+	Preclinical studies conducted in vivo on mice
Fazel, M.F., et al. [43]	Physicochemistry, Nutritional, and Therapeutic Potential of Ficus carica—A Promising Nutraceutical	Review	2024	*F. carica* is a promising medicinal plant for AD due to its polyphenols and bioactive compounds that reduce oxidative stress. Most studies are preclinical, requiring clinical trials for practical use. Cognitive improvements have not been assessed with specific tests.	Dosage not investigated	Preclinical studies conducted in vitro; Preclinical studies conducted in vivo on mice;
Melrose, J., et al. [44]	Natural and Semi-Synthetic Flavonoid Anti-SARS-CoV-2 Agents for the Treatment of Long COVID-19 Disease and Neurodegenerative Disorders of Cognitive Decline	Review	2022	The neuroprotective properties of chalcones and flavones are manifested in the induction of Nrf2 expression, acting on neurogenesis and neuronal differentiation processes. The effects have been evaluated on the induction of gene expression involved in AD neurogenesis.	Dosage not investigated	Preclinical studies conducted in vivo on mice
Saito, S., et al. [45]	Taxifolin: A Potential Therapeutic Agent for Cerebral Amyloid Angiopathy	Review	2021	Preclinical studies show that taxifolin offers greater promise for treating CAA than other drug candidates. Future research on this bioactive flavonoid could lead to new preventive treatments for AD and CAA. Cognitive improvements have not been assessed with specific tests.	Dosage not investigated	Preclinical studies conducted in vivo on mice
Aghababaei, F., et al. [46]	Recent Advances in Potential Health Benefits of Quercetin	Review	2023	Quercetin has shown potential in AD treatment by reducing oxidative stress, modulating neuroinflammation, and targeting senescent cells. Studies indicate that quercetin improves cognitive function in animal models and may reduce AD-related pathologies. However, optimal dosages for AD treatment remain unclear. Further research is needed to establish effective dosages and confirm its therapeutic efficacy in human trials. Cognitive improvements have not been assessed with specific tests.	Dosage not investigated	Preclinical studies conducted in vivo on mice; clinical studies conducted in vivo on humans
Hannan, M.A., et al. [47]	Black cumin (Nigella sativa l.): A comprehensive review on phytochemistry, health benefits, molecular pharmacology, and safety	Review	2021	Black cumin and TQ have been shown to provide significant neuronal protection in AD animal models; they exert a protective action on cholinergic neurons by restoring antioxidant levels and reducing ROS. Cognitive improvement was evaluated based on the reduction in Aβ plaques and the inhibition of the inflammatory response through the downregulation of the NF-κB pathway. Cognitive improvements have not been assessed with specific tests.	10–20 and 40 mg/kg/die of TQ for 14 days to improved cognitive decline in mouse models	Preclinical studies in vitro; preclinical studies conducted in vivo on animal models
Zhou, P., et al. [48]	A Comprehensive Review of Genus Sanguisorba: Traditional Uses, Chemical Constituents and Medical Applications	Review	2021	Sanguisorba minor has a high antioxidant capacity, which can reduce inflammation caused by ROS, a fundamental factor in AD. The activity of S. minor was evaluated based on the production of ROS.	Dosage not investigated	Preclinical studies conducted in vitro; preclinical studies conducted in vivo on rats
Olas, B. [49]	The Current State of Knowledge about the Biological Activity of Different Parts of Capers	Review	2023	Hydroalcoholic extracts from *C. spinosa* fruits and leaves have the potential to reduce inflammation in AD animal models by regulating the genes involved. A decrease in the concentrations of inflammatory mediators, osteoclasts, and chondrocytes was observed.	Phenolic extract of seed of *C. spinosa*: 50–100 and 200 mg/kg for 8 weeks in animal models increases antioxidant action	Preclinical studies in vitro; preclinical studies conducted in vivo on animal models
Balakrishnan, R., et al. [50]	Development of dietary small molecules as multi-targeting treatment strategies for Alzheimer’s disease	Review	2024	Dietary small molecules inhibit AChE activity, preventing acetylcholinesterase breakdown, Aβ plaque deposition, and the onset of neuronal inflammation; inhibiting tau hyperphosphorylation; and alleviating synaptic dysfunction. Cognitive improvements have not been assessed with specific tests.	Dosage not investigated	Preclinical studies conducted in vitro; preclinical studies conducted in vivo on mice and rats; clinical studies conducted on human
Saha, P., et al. [51]	Neuroprotective, Anti-Inflammatory and Antifibrillogenic Offerings by Emodin against Alzheimer’s Dementia: A Systematic Review	Review	2024	Emodin shows potential as an antifibrillogenic, antineuroinflammatory, and neuroprotective agent against AD. Cognitive improvements have not been assessed with specific tests.	50 mg/kg of body weight/die of emodin	Preclinical studies conducted in vitro; preclinical studies conducted in vivo on mice
Andrade, S., et al. [52]	Therapeutic Potential of Natural Compounds in Neurodegenerative Diseases: Insights from Clinical Trials	Review	2023	Vitamin supplementation (C, E, and B group) can reduce the levels of Aβ peptide and amyloid plaque deposition. DHA exerts a neuroprotective action and acts against the deposition of the Aβ peptide. The levels of alpha and beta in the cerebrospinal fluid have been evaluated.	20 µg/die of bryostatin-1; 400 µg/die of Huperzine-A; 150 mg of Homotaurin twice a day; 2 mg/die of melatonin	Clinical studies conducted in vivo on humans
Schouten, M., et al. [53]	Cannabidiol and brain function: current knowledge and future perspectives	Review	2024	In animal models, cannabidiol administration suppressed the neuroinflammatory response induced by amyloid deposits ad hippocampal neurogenesis, delaying the progression of AD; cannabidiol also improved cognitive performance. Cannabidiol may address AD healthcare needs by interacting with various receptors (CB1, CB2, and 5-HT1A) and influencing ion channels. Cognitive improvements have not been assessed with specific tests.	20 mg/kg of body weight/die	Preclinical studies conducted in vitro; preclinical studies conducted in vivo on mice and rats
Szymczak, J., et al. [54]	Fisetin—In Search of Better Bioavailability—From Macro to Nano Modifications: A Review	Review	2023	Physetin decreases Aβ accumulation, BACE-1 expression, and the hyperphosphorylation of the tau protein at Ser 413; activates p-PI3K, p-Akt (Ser 473), and p-GSK3 (Ser 9) expression, and protects from neuroinflammation. The action of physetin has been evaluated based on the molecular processes underlying AD neurogenesis.	Dosage not investigated	Preclinical studies conducted in vivo on mice
Schepici, G., et al. [55]	Efficacy of Sulforaphane in Neurodegenerative Diseases	Review	2020	SFN is capable of inhibiting Aβ aggregation, Tau hyperphosphorylation, and oxidative stress; additionally, it reduces neuroinflammatory inflammation modulated by TNF-α and IL-1β. Cognitive improvements have not been assessed with specific tests.	Dosage not investigated	Preclinical studies conducted in vitro; preclinical studies conducted in vivo on mice and rats
Bertoni, C., et al. [56]	The role of Alpha-Linolenic Acid and other Polyunsaturated Fatty Acid in Mental Health: A Narrative Review	Review	2024	Low PUFA levels were observed in AD patients, suggesting that supplementation may offer neuroprotective effects. ALA supplement resulted in improved verbal skills. Cognitive improvements have not been assessed with specific tests.	2 g /die of ALA	Clinical studies conducted in vivo on humans
Kumar, S., et al. [57]	Nutritional neurology: unraveling cellular mechanisms of natural supplements in brain health	Review	2024	Nutritional supplementation can support brain health in AD. ϖ-3 fatty acids promote neuronal plasticity, but excessive intake may increase oxidative stress. A multinutrient approach, including DHA, EPA, B vitamins, and antioxidants, helps slow cognitive decline. The ketogenic diet enhances brain metabolism and reduces Aβ accumulation. Polyphenols, such as resveratrol, have neuroprotective effects, while intermittent fasting supports cognitive function and mitigates neurodegeneration. Cognitive improvements have not been assessed with specific tests.	Dosage not investigated	Clinical studies conducted in vivo on humans
Chimakurthy, A.N., et al. [58]	A Systematic Review of Dietary Supplements in Alzheimer’s Disease	Review	2023	Some supplements are used alongside anticholinesterase inhibitors, which have a beneficial effect. The combination of different compounds has shown improvements in daily and mnemonic activities. Cognitive improvement was evaluated using WMS, LDS, ADL, MNA-S,F and ADAS-cog.	100 mg/die of Phosphatidylserine in combination with 80 mg/die of phosphatic acid; 3000 UI/die of Vitamin D_2_; 125 mL/die of Suvenaid for mild AD; formulation of 400 µg of FA; 6 µg of vitamin B_12_; 30 UI of Vitamin E, 600 mg of N-acetylcysteine, 400 mg of S-adenosylmethionine and 500 mg of Acetyl LC for mild AD	Clinical studies conducted in vivo on humans
Cunnane, S.C., et al. [59]	Mild cognitive impairment: when nutrition helps brain energy rescue—a report from the EuGMS 2020 Congress	Review	2021	Ketone bodies are capable of mediating DNA acetylation processes at the neuronal level; moreover, they are effective in reducing inflammation in neurodegenerative diseases. The assessment of cognitive improvement was conducted using the MMSE.	30 g/die of kMCT	Clinical studies conducted in vivo on humans
Soto-Mota, A., et al. [60]	Why a D-β-hydroxybutyrate monoester?	Review	2020	The βHB monoester shows potential as a treatment for AD. Its ability to rapidly adjust βHB levels in the blood also aids in studying ketone metabolism in AD. Cognitive improvements have not been assessed with specific tests.	357–714 mg/kg of body weight/die of βHB	Clinical studies conducted in vivo on humans
Gil Martínez, V., et al. [61]	Vitamin Supplement and Dementia: A Systematic Review	Review	2022	This systematic review suggests that B complex vitamins, especially FA, may help delay or prevent cognitive decline, particularly in AD. Ascorbic acid and high-dose vitamin E showed positive effects on cognitive performance, but due to the limited number of studies, there is not enough evidence to strongly recommend them. The effects of vitamin D supplementation on cognition were inconsistent across trials, making it difficult to establish its definitive benefits in AD. Cognitive function measured by cognitive scales like MMSE, ADAS-Cog, WAIS-RC, TMT, MocA, ADL-Score, FAB-Score, or CDR.	800 µg/die of FA 500–1000 mg/die of Ascorbic Acid in combination with 400 IU/die of Vitamin E 800–1000 UI/die of Vitamin D	Clinical studies conducted in vivo on humans
Puga, A.M., et al. [62]	Effects of Supplementation with Folic Acid and Its Combinations with Other Nutrients on Cognitive Impairment and Alzheimer’s Disease: A Narrative Review	Review	2021	The combination of folic acid and DHA showed a decrease in the blood levels of Aβ biomarkers (Aβ40 e Aβ42) and Hcy. Cognitive improvements were assessed using the Digit Span subtest of the WAIS-RC, MMSE, and the Social Behavior Score.	400 µg/die of FA	Clinical studies conducted in vivo on humans
Diaz, G., et al. [63]	Effect of B-vitamins supplementation on cognitive decline in patients with Alzheimer’s disease. A systematic review and meta-analysis	Review	2023	Evidence suggests that B vitamin supplementation has little to no effect on global cognitive decline in AD, as measured by the ADAS-Cog, CDR, and MMSE. The supplementation of two years of folic acid, B6, and B12 improved the cognitive performance in patients with cognitive impairment. Cognitive improvement was evaluated using the ADAS-Cog, CDR, and MMSE.	Dosage not investigated	Clinical studies conducted in vivo on humans
Zhang, L., et al. [64]	A Comparative Study Evaluating the Effectiveness of Folate-Based B Vitamin Intervention on Cognitive Function of Older Adults under Mandatory Folic Acid Fortification Policy: A Systematic Review and Meta-Analysis of Randomized Controlled Trials	Review	2024	Folate-based B vitamin supplementation significantly improves cognitive function in older adults, particularly in regions without mandatory food fortification. However, in areas with mandatory FA fortification, no significant effects were observed, suggesting that fortification may reduce the benefits of additional supplementation. In regions without fortification, older adults may suffer from folate deficiency, making FA supplementation crucial for improving cognitive health and offering significant public health benefits. Cognitive improvements have not been assessed with specific tests.	Dosage not investigated	Clinical studies conducted in vivo on humans
Dos Santos, S.M., et al. [65]	Mitochondrial Dysfunction and Alpha-Lipoic Acid: Beneficial or Harmful in Alzheimer’s Disease?	Review	2019	Intake of α-LA increases the expression of cAMP, leading to the inhibition of pro-inflammatory cytokines and an increase in IL-10, reducing inflammation. The assessment of the improvement in AD patients was conducted based on the epigenetic regulation of inflammation-related genes.	Dosage not investigated	Clinical studies conducted in vivo on humans
Tòth, F., et al. [66]	Natural molecules and neuroprotection: Kynurenic acid, panthenine and α-lipoic acid	Review	2020	Kynurenic acid, pantethine, and α-lipoic acid have neuroprotective properties that can contribute to the treatment of neurodegenerative diseases like AD. Due to their ability to protect neurons and improve cognitive functions, these compounds are promising candidates for neuroprotective therapies. Cognitive improvement was evaluated using the MMSE, ADAS-cog, and cognitive subscale.	600 mg LA in combination with acetylcholinesterase inibitore.	Clinical studies conducted in vivo on humans
Burckhardt, M., et al. [67]	Souvenaid for Alzheimer’s disease	Review	2020	Souvenaid, a dietary supplement designed to support brain synapse function in AD, was tested in three randomized, placebo-controlled trials. Specifically, there was a slight, non-significant improvement in prodromal AD after 24 months, but no significant difference in mild-to-moderate AD dementia after 24 weeks. Adverse effects were low, and the data were insufficient to draw conclusions on any direct link to the supplement. Cognitive improvement was evaluated using the ADAS-cog, MMSE, RAVLT, NTB, ADCS-ADL, GBS-ADL, CDR-SOB, and CIBIC-Plus.	Dosage not investigated	Clinical studies conducted in vivo on humans
Gojon, G., et al. [68]	SG1002 and Catenated Divalent Organic Sulfur Compounds as Promising Hydrogen Sulfide Prodrugs	Review	2020	H_2_S and glutathione are therapeutic agents in degenerative diseases with the ability to modulate ROS production and decrease inflammatory states. Inflammation markers have been evaluated.	Dosage not investigated	Clinical studies conducted in vivo on humans
Ebrahimi, A., et al. [69]	Involvement of Coenzyme Q10 in Various Neurodegenerative and Psychiatric Diseases	Review	2023	CoQ10 acts by decreasing oxidative stress markers such as protein carbonylis; CoQ10 supplementation has protective effects against brain atrophy. A decrease in the production of Aβ 42 and a reduction in oxidative stress markers have been observed.	Dosage not investigated	Clinical studies conducted in vivo on humans
Al-Dhuayana, I.S. [70]	Biomedical role of L-carnitine in several organ systems, cellular tissues, and COVID-19	Review	2022	The structure of LC improves and preserves cognitive performance and contributes to improved cognitive aging over time. Multiple controlled human clinical trials using LC have provided evidence that this substance can improve cognitive function. Cognitive improvements have not been assessed with specific tests.	Dosage not investigated	Clinical studies conducted in vivo on humans
Wang, W., et al. [71]	L-Carnitine in the treatment of Psychiatric and Neurological Manifestations: A Systematic Review	Review	2024	LC has shown potential in treating AD. Despite mixed findings, LC is considered a safe, affordable option for alleviating AD symptoms, warranting further research to confirm its effectiveness and understand its mechanisms. Cognitive improvements have not been assessed with specific tests.	2 g/die or 3 g/die of LC	Clinical studies conducted in vivo on humans
Mishra, A., et al. [72]	Potential role of herbal plants and β sitosterol as a bioactive constituent in circumventing Alzheimer’s Disease	Review	2024	Several dietary strategies show potential in AD management. *Melissa officinalis* extract, rich in rosmarinic acid, has been clinically tested for slowing disease progression, while gastrodin demonstrated neuroprotective effects in patients undergoing cardiopulmonary bypass. These findings highlight the role of natural compounds and metabolic interventions in neuroprotection. Cognitive improvements have not been assessed with specific tests.	Dosage not investigated	Preclinical studies conducted in vitro; preclinical studies conducted in vivo on mice
Constantinescu, T., et al. [73]	Resveratrol as a privileged molecule with antioxidant activity	Review	2023	Resveratrol crosses the blood–brain barrier, reducing neuronal loss and oxidative stress in AD by inhibiting Aβ formation and enhancing lipid metabolism. In stroke, resveratrol activates the AMPK pathway, reducing ROS, oxidative stress, and apoptosis. Its antioxidant effects are mediated by Nrf2, boosting antioxidant gene expression. Cognitive improvements have not been assessed with specific tests.	3 g/ die of Resveratrol	Preclinical studies conducted in vitro; preclinical studies conducted in vivo
Ghafouri-Fard, S., et al. [74]	Disease-associated regulation of gene expression by resveratrol: Special focus on the PI3K/AKT signaling pathway	Review	2022	Resveratrol shows promise in AD by reducing Aβ toxicity, inhibiting tau phosphorylation, and improving neurological function. It regulates autophagy and apoptosis via the Akt/mTOR pathway. It also modulates immune responses and ROS formation, with potential in cancer treatment. Cognitive improvements have not been assessed with specific tests.	3–5 mg/die of Resveratrol	Clinical studies conducted on human
Minich, D.M., et al. [75]	Is Melatonin the “Next Vitamin D”?: A Review of Emerging Science, Clinical Uses, Safety, and Dietary Supplements	Review	2022	Melatonin supplementation is suggested to improve sleep and neurotransmission, reducing sundowning, and potentially slowing AD progression by protecting neurons from Aβ damage and enhancing amyloid clearance through the glymphatic fluid. Cognitive improvement was evaluated using the MMSE and the Sleep Disorders Index.	Dosage not investigated	Clinical studies conducted in vivo on humans
Givler, D., et al. [76]	Chronic Administration of Melatonin: Physiological and Clinical Considerations	Review	2023	Melatonin, commonly used to treat sleep disorders and cognitive decline, may help alleviate the symptoms of AD. While it is considered safe in low to moderate doses, further research is needed to fully establish its long-term therapeutic effects on AD. Cognitive improvements have not been assessed with specific tests.	3–5 mg/die of melatonin	Preclinical studies conducted in vitro; clinical studies conducted in vivo on humans
Song, X., et al. [77]	Caffeine: A Multifunctional Efficacious Molecule with Diverse Health Implications and Emerging Delivery Systems	Review	2024	Caffeine may offer neuroprotective effects in AD by blocking A2A adenosine receptors, which helps reduce neuroinflammation and protect against neuronal damage. Studies suggest that moderate caffeine consumption can help prevent cognitive decline and reduce amyloid plaque formation, key features in AD. Cognitive improvements have not been assessed with specific tests.	5 cups of coffee/die	Preclinical studies conducted in vivo on mice; clinical studies conducted in vivo on humans
Talebi, M., et al. [78]	Molecular mechanism-based therapeutic properties of honey	Review	2020	Honey and its compounds, especially chrysin, show potential in AD treatment by modulating oxidative stress, reducing neuroinflammation, and protecting neurons from Aβ toxicity, thus offering neuroprotective benefits. Cognitive improvements have not been assessed with specific tests.	Dosage not investigated	Preclinical studies conducted in vitro; preclinical studies conducted in vivo on mice and rats; clinical studies

Abbreviations: 5-HT, Serotonin; Aβ, amyloid beta; AchE, acetylcholinesterase; AD, Alzheimer’s disease; ADAS-cog, Alzheimer’s Disease Assessment Scale–Cognitive Subscale; ADCS, Alzheimer’s Disease Cooperative Study; ADL, Activities of Daily Living; Akt, Protein kinase B; α-LA, α-lipoic acid; ALA, alpha-linolenic acid; AMPK, 5’ adenosine monophosphate-activated protein kinase; BACE-1, Beta-Secretase 1; βHB, monoester β-hydroxybutyrate monoester; CAA, cerebral amyloid angiopathy; cAMP, cyclic AMP; CB, Cannabinoid; CDR, Clinical Dementia Rating Scale; CDR-SOB, Clinical Dementia Rating Scale Sum of Boxes; CIBIC-Plus, Clinician’s Interview-Based Impression of Change Plus caregiver input; CoQ10, coenzyme Q10; DHA, docosahexaenoic acid; EPA, eicosapentaenoic acid; FA, Folic Acid; FAB, Frontal Assessment Battery; GBS, Gottfries-Brane–Steen Scale; GSK3, Glycogen synthase kinase-3; Hcy, homocysteine; H_2_S, hydrogen sulfide; IL, Interleukine; kMCT, Ketogenic medium-chain triglycerides; LC, L-carnitine; LDS, Laboratory Decision System; MMSE, Mini Mental State Examination; MNA-SF, Mini Nutritional Assessment Short Form; MocA, Montreal Cognitive Assessment; mTOR, mechanistic target of rapamycin; NAD+, Nicotinamide-adenine dinucleotide; NFκB, nuclear factor kappa-light-chain-enhancer of activated B cells; NR, Nicotinamide riboside; Nrf2, Nuclear factor erythroid 2-related factor 2; NTB, Neuropsychological Test Battery; PARylation, Protein poly ADP-ribosylation; PI3K, Phosphoinositide 3-kinases; PUFA, polyunsaturated fatty acid; RAVLT, Rey Auditory Verbal Learning Test; ROS, reactive oxygen species; Ser, Serine; SFN, sulforaphane; TMT, Trail Making Test; TNF-α, Tumor necrosis factor-alpha; TQ, thymoquinone; WAIS-RC, Wechsler Adult Intelligence Scale—Revised; WMS, Wechsler Memory Scale; ω-3, omega-3.

**Table 3:** Main characteristics of all articles on the safety and efficacy of prebiotics and probiotics supplementation in AD.

Authors	Title	Type of Article	Date	Efficacy	Safety	Type of Study
Ji Jang, H., et al. [79]	A Narrative Review on the Advance of Probiotics to Metabiotics	Mini-review	2024	Certain bacterial species, such as *L. reuteri*, *L. rhamnosus*, and *B. fragilis*, produce SCFAs that regulate the cholinergic signaling and inflammatory processes of the vagus nerve, reducing AD symptoms. *L. reuteri* is capable of promoting IL-10 synthesis and increasing dopamine levels in the blood. In mouse models, efficacy was evaluated through the reduction in Aβ-induced inflammation.	Dosage not Investigated.	Preclinical studies conducted in vivo on mice
Yuan, C., et al. [80]	Review of microbiota gut–brain axis and innate immunity in inflammatory and infective diseases	Review	2023	An increase in *Verrucomicrobia* and *Proteobacteria*, along with a decrease in *Ruminococcus* and *Butyricicoccus*, is correlated with AD. The decrease in SCFA levels highlights how metabolic pathways are linked to AD. Cognitive improvements have not been assessed with specific tests.	Dosage not Investigated.	Preclinical studies conducted in vitro; preclinical studies conducted in vivo on mice and rats; clinical studies conducted on humans
Novik, G., et al. [81]	Beneficial microbiota. Probiotics and pharmaceutical products in functional nutrition and medicine	Review	2020	Reviewed studies show that probiotics, especially LAB and *Bifidobacteria*, benefit host health by modulating immune responses and impacting pathogenic microorganisms. Recent findings suggest they may also aid in AD treatment and prevention. Cognitive improvements have not been assessed with specific tests.	12-week supplementation of probiotic milk composed of *L. acidophilus*, *L. casei*, *B. bifidum*, and *L. fermentum.* No dosage was specified.	Clinical studies conducted in vivo on humans
Lv, T., et al. [82]	Probiotics treatment improves cognitive impairment in patients and animals: A systematic review and meta-analysis	Review	2021	Probiotics have a significant positive effect on cognitive function, particularly in individuals with cognitive impairments, but not in healthy individuals, where they may even have a negative impact. Given their strain-specific effects and variability in cognitive conditions, a personalized approach is recommended for cognitively impaired individuals. Cognitive improvements have not been assessed with specific tests.	Dosage not investigated.	Preclinical studies conducted in vivo on animals models; clinical studies conducted in vivo on humans
Martinez-Guardado, I., et al. [83]	The Therapeutic Role of Exercise and Probiotics in Stressful Brain Conditions	Review	2022	Physical exercise can promote angiogenesis, improve cerebral blood flow, and reduce the production of IL-1β and TNF-α. The use of prebiotics stimulates SCFA production, decreasing ROS production and increasing NO production, thereby promoting antioxidant processes. Cognitive improvements have not been assessed with specific tests.	1–2 × 10^10^ CFU of probiotics for 12–24 weeks.	Preclinical studies conducted in vivo on mice; clinical studies conducted in vivo on humans
Den, H., et al. [84]	Efficacy of probiotics on cognition, and biomarkers of inflammation and oxidative stress in adults with Alzheimer’s disease or mild cognitive impairment—a meta-analysis of randomized controlled trials	Review	2020	The microbiota are crucial in the pathogenesis of AD through the MGB axis, with evidence showing that restoring intestinal homeostasis can positively slow AD progression. Cognitive improvements have not been assessed with specific tests.	Dosage not Investigated.	Clinical studies conducted in vivo on humans

Abbreviations: AD, Alzheimer’s disease; IL, Interleukine; LAB, lactic acid bacteria; MGB, gut-microbiota–brain axis; NO, Nitric Oxide; ROS, reactive oxygen species; SCFA, short-chain fatty acids; TNF-α, Tumor necrosis factor-alpha.

## Abbreviations

The following abbreviations are used in this manuscript:

5-HT	Serotonin
α-LA	α-lipoic acid
Aβ	Beta amyloid
AChE	Acetylcholinesterase
AD	Alzheimer’s diseas
Akt	Protein kinase B
ALA	α-linolenic acid
AMPK	AMP-activated protein kinase
APP	Amyloid precursor protein
βHB	D-β-hydroxybutyrate
BACE1	Beta-secretase 1
BBB	Blood–brain barrier
BuChE	Butyrylcholinesterase
CAA	Cerebral amyloid angiopathy
cAMP	Cyclic AMP
CAT	Catalase
COX-2	Cyclooxygenase-2
DHA	Docosahexaenoic acid
EGCG	Epigallocatechin gallate
EPA	Eicosapentaenoic acid
GABA	γ-aminobutyric acid
GM	Gut microbiota
GSH-Px	Glutathione peroxidase
GSK3	Glycogen synthase kinase 3
H_2_S	Hydrogen sulfide
Hcy	Homocysteine
HO-1	Heme oxygenase
IL	Interleukin
iNOS	Nitric oxide synthases
kMCT	Ketogenic medium-chain triglyceride
LA	linoleic acid
LC	L-carnitine
LPS	Lipopolysaccharide
LRR	Leucine-rich repeat
MAO	Monoamine oxidase
MAPK	Mitogen-activated protein kinase
MedDiet	Mediterranean diet
mTOR	Mechanistic target of rapamycin
NADPH	Nicotinamide adenine dinucleotide phosphate
NFκB	Nuclear factor kappa-light-chain-enhancer of activated B cells
NFT	Neurofibrillary tangles
NLRP3	Pyrin domain-containing protein 3
NMDA	N-methyl-D-aspartate
NO	Nitric oxide
NOD	Nucleotide-binding domain
Nrf2	Nuclear factor erythroid 2
NSAIDs	Non-steroidal anti-inflammatory drugs
PI3K	Phosphoinositide 3-kinases
PTEN	Phosphatase and tensin homolog
PUFA	Polyunsaturated fatty acid
ROS	Reactive oxygen species
SIRT	Sirtuin
SCFA	Short-chain fatty acid
SOD	Superoxide dismutase
TLR4	Toll-like receptor 4
TMAO	Trimethylamine N-oxide
TNF-α	Tumor necrosis factor alpha
ϖ-3	Omega-3
